# Navigating the local foodscape: qualitative investigation of food retail and dietary preferences in Kisumu and Homa Bay Counties, western Kenya

**DOI:** 10.1186/s12889-022-13580-4

**Published:** 2022-06-14

**Authors:** Rosemary M. Musuva, Louise Foley, Pamela Wadende, Oliver Francis, Charles Lwanga, Eleanor Turner-Moss, Vincent Were, Charles Obonyo

**Affiliations:** 1 Center for Global Health Research, Kenya Medical Research Institute, P. O. Box 1578, Kisumu, 40100 Kenya; 2 MRC Epidemiology Unit, Institute of Metabolic Science, Cambridge Biomedical Campus, University of Cambridge, P.O Box 285, Cambridge, CB2 0QQ UK; 3 Faculty of Education and Human Resources, Kisii University, PO Box 408, Kisii, 40200 Kenya; 4 Adaptive Management and Research Consultants (AMREC) Africa, P.O Box 5022, Kisumu, 40141 Kenya

**Keywords:** Dietary preference, Foodscape, Non-communicable diseases, Western Kenya, Ecological model

## Abstract

**Introduction:**

Non-communicable diseases have risen markedly over the last decade. A phenomenon that was mainly endemic in high-income countries has now visibly encroached on low and middle-income settings. A major contributor to this is a shift towards unhealthy dietary behavior. This study aimed to examine the complex interplay between people’s characteristics and the environment to understand how these influenced food choices and practices in Western Kenya.

**Methods:**

This study used semi-structured guides to conduct in-depth interviews and focus group discussions with both male and female members of the community, across various socioeconomic groups, from Kisumu and Homa Bay Counties to further understand their perspectives on the influences of dietary behavior. Voice data was captured using digital voice recorders, transcribed verbatim, and translated to English. Data analysis adopted an exploratory and inductive analysis approach. Coded responses were analyzed using NVIVO 12 PRO software.

**Results:**

Intrapersonal levels of influence included: Age, the nutritional value of food, occupation, perceived satiety of some foods as opposed to others, religion, and medical reasons. The majority of the participants mentioned location as the main source of influence at the community level reflected by the regional staple foodscape. Others include seasonality of produce, social pressure, and availability of food in the market. Pricing of food and distance to food markets was mentioned as the major macro-level influence. This was followed by an increase in population and road infrastructure.

**Conclusion:**

This study demonstrated that understanding dietary preferences are complex. Future interventions should not only consider intrapersonal and interpersonal influences when aiming to promote healthy eating among communities but also need to target the community and macro environments. This means that nutrition promotion strategies should focus on multiple levels of influence that broaden options for interventions. However, government interventions in addressing food access, affordability, and marketing remain essential to any significant change.

**Supplementary Information:**

The online version contains supplementary material available at 10.1186/s12889-022-13580-4.

## Introduction

People must eat to live, but beyond this basic biological function, food forms an integral part of our daily lives. Food consumption has evolved into a multifaceted social instrument. It is a principal social and cultural activity that people enjoy for aesthetic or communal reasons while connecting people across cultural boundaries [1]. However, dietary behaviors have increasingly become a cause for concern because of their associations with ill health and mortality. Analysis of the Global Burden of Disease Study 2010 [2] shows that dietary factors are the most important factors that undermine health and well-being. It is recognized that malnutrition, including undernutrition, micronutrient deficiencies, overweight, and obesity, as well as non-communicable diseases (NCDs) resulting from unhealthy diets, have high social and economic costs for individuals, and families, communities, and governments [3].

Globally, non-communicable diseases (NCDs) are the leading cause of death. According to WHO estimates, these diseases contributed to 36 million deaths globally in 2008, accounting for 63% of 57 million total deaths [4]. It is also projected that NCDs will account for an increasing absolute number and proportion of worldwide deaths, rising to about 70% of deaths in 2030 [4]. About 80% of deaths related to NCDs occur in low- and middle-income countries (LMICs) [5]. In many ways, this shift is a continuation of large-scale changes that have occurred over time.

Economic development in LMICs together with recent technological innovations and modern marketing techniques have modified dietary preferences. This has led to major changes in the composition of diet which contributes to the prevalence of NCDs [6]. Specifically, there has been a shift towards high fat, refined carbohydrate, and a low-fiber diet. These dietary changes and the related increase in diet-related diseases are intensified in Africa by the rapid increase in urbanization. [7]. As seen in other countries, the rise of fast-food restaurants and the influx of sugar-sweetened beverages are at an all-time high [8]. This is further exacerbated by the transformation of the local food environment with supermarkets infiltrating the inner city and even rural neighborhoods [9], potentially replacing traditional wet markets offering fresh food and produce [10].

This is particularly true in Kenya where the middle-class boom has resulted in a larger market for processed foods from supermarkets and a decline in fresh foods available in traditional markets. Supermarkets in urban Kenya have risen from a tiny niche a half-decade ago to a fifth of food retail, spreading well beyond the richer consumers to derive more than a third of their sales and half of their customers from low income and poor consumers. The United Nations Sustainable Development Goal (SDG) 2 seeks “to end hunger, achieve food and nutrition security, improve nutrition, and promote sustainable agriculture” [11]. Against this backdrop, “improving knowledge and understanding about food environments – including the who, what, when, where, why, and how of food acquisition and consumption – will be key to addressing malnutrition in all its forms” [12]. Food choice is a complex phenomenon, affected by many interrelated factors described by various levels of influence. This study, therefore, sought to explore the influencers of dietary choices and preferences across three levels of influence -– interpersonal, community, and national/policy among residents of Homa Bay and Kisumu Counties in Western Kenya. This evidence is essential to support the designing of policies and interventions that appropriately leverage agricultural biodiversity, in concert with components of other food systems, to address the multiple burdens of malnutrition in LMICs.

The analysis described here draws on baseline findings from a larger ongoing mixed-method natural experimental study evaluating the impacts of a new hypermarket (supermarket combined with a department store) on dietary behavior and the local foodscape in Western Kenya [13]. The main aim of this analysis was to explore the relationship between food retail and dietary behavior among members of the community in Western Kenya. The study was conducted in two study sites: The intervention site (Kisumu, where the hypermarket is being developed) and a comparison site (Homa Bay, an equally cosmopolitan town but without a hypermarket).

## Materials and methods

### Conceptual framework

The ecological model was adopted in the formulation of data collection tools. This model recognizes the complex interplay that exists between an individual and the various levels of interaction with the environment [14]. This was particularly appropriate in the study context of a middle-income country facing rapid economic growth and a shift in culture alongside changing local foodscapes. The choice of food could be influenced at multiple levels. Individual characteristics such as level of education, knowledge or perception of healthy food, and personal preferences could shape choices. Using the community as the second level of interaction seeks to understand a community’s norms and culture and the role they play in the general health and wellbeing of its people. Examples can be drawn from taboo foods, communal sporting activities, groups, or organizations in the community that promotes or hinder healthy dietary behavior. In addition, the enabling or limiting factors at the national level could also influence the local foodscape: for instance, the level of tax on certain foods, levies on fast food restaurants, advertisements on highly processed foods, or policies on the location of malls and wet markets.

### Study site

The study was conducted in Kisumu and Homa Bay counties, in Western Kenya. These settings have a population of 1,155,574, and 1,131,950, respectively [15]. Two study areas were defined: the hypermarket intervention area (Mamboleo, Kisumu) and a comparison area with no hypermarket (Sofia, Homa Bay). These areas were delineated using existing spatial census data, field visits, and local knowledge. A 2 km radial buffer was drawn around the hypermarket and matched according to population density with a 2 km radial buffer around Sofia as the landmark in the comparison area. Both sites display similar food retail, socioeconomic (both lower and higher), and topographical characteristics. Dominant socioeconomic activities in both sites include fishing, small-scale farming, and the steady growth of both Counties leading to an increase in consumers seeking convenient shopping avenues such as supermarkets and upscale grocery vendors.

### Study design

This was a cross-sectional qualitative study involving members of households who participated in the initial quantitative household survey [16], purposively sampled for follow on qualitative data collection.

### Selection of participants

With the establishment of primary health care networks (PCNs) and subsequent implementation of the Kenya Primary Health Care Strategic Framework 2019 – 2024 [17], the study team worked closely with the departments of health in both Kisumu and Homa Bay Counties which have a functional community health unit [18]. Through this system, the community health volunteers (CHVs) who are at the first level of care and link households to health care facilities were recruited. Twenty community health volunteers working within a 2 km radius of the Lake Basin Mall and Sophia area assisted in generating lists of 2000 households [13]. A stratified sampling technique (probability proportionate to size) was then used to randomly sample by household SES (low, middle, and high – classification described in more detail below), distance (within 0.5 km, 1 km, and 2 km from the mall and Sophia area) and quadrant (NE, NW, SE, and SW). Based on these criteria, the final sample comprised 200 households estimated from the main protocol which assumed a 5% household food expenditure share, 80% power, 95% confidence interval, and a 30% attrition rate [19]. From these households, face-to-face questionnaires were administered. Finally, those who consented to participate further in the qualitative arm of the study after completing the questionnaires were then randomly selected from the various quadrants. Phone calls were later made to these individuals to confirm their availability and agree on the time, date, and venue for the FGDs. For the in-depth interviews, participants were purposefully selected from a list of stakeholders previously engaged in the community entry exercise of the study. IDIs took place in the interviewee’s office or board rooms within the office buildings.

### Qualitative inquiry methods

Using the saturation model for qualitative data [20] ceasing additional data collection once which focuses on when the ability to obtain additional new information has been attained, four focus group discussions (FGD) each with a maximum of 12 participants were conducted in each county (for eight FGD total) stratified by gender, and social-economic status: i) Males from low socio-economic status households ii) Males from high socio-economic status households iii) Females from low socio-economic status households iv) Females from high socio-economic status households. The FGDs and in-depth interviews (IDIs) were conducted in either Swahili or Dholuo after consensus from the participants.

The focus group discussion guides (Additional file 1: Appendix 1) explored sources of food and reasons for their preference as well as household food staples and their reasons for this preference. 20 stakeholders were identified for the IDIs in Homabay County. The IDI discussion guide (Appendix 2) focused on similar themes to the FGD guides. The IDIs conducted in Kisumu focused on the upcoming hypermarket and the stakeholder’s involvement and were therefore excluded from this analysis.

The study recruited experienced qualitative data collectors of bachelor’s degree level. Prior to the commencement of the study, a three-day training was conducted on understanding the study aims. A refresher training was also offered on IDI and focus group discussion techniques and the discussion guides. After the training, the tools were piloted in both sites and necessary adjustments were made. Both the FGDs and IDIs were conducted by a moderator who was in charge of steering the conversation, and a notetaker who took notes verbatim and in addition captured the non-verbal cues during the discussion. The interviews took an average of one and a half hours each for the FGDs and close to forty minutes for the IDIs. They were conducted in either Swahili or Dholuo. These discussions were held at local venues such as classrooms, community, and church halls and offices. The discussions were recorded using an audio recorder.

### Data analysis

The thematic analysis used in this study was informed by the blended approach to coding described by Graebner [21]. The audio recordings were transcribed verbatim into Microsoft Word. The transcripts were then translated into English and back-translated to ensure no meaning was lost. Transcripts were checked against the note-takers details notes and audio recordings to ensure they were a true reflection of the proceedings therefore not warranting correction from the study participants. Three experts first read the transcripts iteratively to generate ideas through data immersion. Initial codes were then systematically generated within and across the full dataset. Themes were identified among the codes, and these were discussed and modified until consensus was reached. Saturation was reached before all the transcripts were analyzed because no new codes were identified when coding the last interview. The final themes were checked against the coded extracts and the full dataset. Once key themes had been identified, the final stage included defining which data qualities each theme captured, and a detailed analysis was written to describe the theme, including relevant sub-themes. Finally, the research team worked collaboratively to develop an intercoder agreement [22]. Discrepancies were resolved on a case-by-case basis until a full agreement was reached. The coding tree is provided as a supplemental file.

## Results

### Socio-demographic characteristics of the study participants

The study recruited 33 and 38 participants in Kisumu and Homa Bay Counties respectively giving a total of 71 respondents. This group constituted of males and females aged 20–69 years from different socio-economic groups. The majority of the participants constituted those between ages 30–39, 31.6% in Homa Bay while in Kisumu those between 40–49 formed the majority by 27.3%. On both sites, > 50% of participants were married and had attained at least primary school level education. A summary of the sociodemographic details of the focus group discussion participants held with community members in Kisumu and Homa Bay Counties is presented in Table 1. A summary of in-depth interview participants is provided in Table 2.

**Table 1:** Sociodemographic characteristics of FGD participants in Kisumu and Homa Bay

	**Kisumu County**		**Homa Bay County**	
**Description**	**Frequency** **(*n* = 33)**	**Percentage (%)**	**Frequency (*n* = 38)**	**Percentage (%)**
**Gender**				
Female	15	45.5	21	55.3
Male	18	54.5	17	44.7
**Age (Years)**				
20–29	8	24.2	9	23.7
30–39	6	18.2	12	31.6
40–49	9	27.3	9	23.7
50–59	7	21.2	6	15.8
60–69	3	0.1	2	5.3
**Educational Level**				
None	1	0.03	0	0
Primary	22	66.7	23	60.5
Secondary	6	18.2	9	23.7
A-Level/college	3	0.99	6	15.8
Didn’t disclose	1	0.03	0	0
**Occupation**				
Formal employment	2	6.06	6	15.8
Business	17	51.5	11	28.9
Semi-skilled labor	10	30.3	6	15.8
Farmer/Agriculture	1	3.03	8	21.1
Unemployed	3	9.09	6	15.8
Didn’t disclose	0	0	1	2.6
**Marital status**				
Married	27	81.8	30	78.9
Single	3	0.09	2	5.2
Widow	3	0.09	6	15.8

**Table 2:** In-depth Interview participants

Role	Number
Trade	9
Health	2
Environment	2
Administration	2
Agriculture	2
Faith	1
Education	2
Total	20

### Interpersonal influences

#### Choice of food

Participants cited perceived satiety of some food types, age, occupation, taste, preference, and medical reasons as some of the influencing factors on what they would eat. The amount of money one has was mentioned by most of the participants.

“*People who engage in strenuous activities take heavy foods, they like Githeri (a mix of maize and beans) sweet potatoes and all the rest…You will mostly find that those who go for construction work when you go to the construction site you will find that they do not eat light food*.” ( Boda Boda rider, IDI Respondent, Homabay).

*“… It will depend on the pocket. You know here the price of tilapia. So, you will have to buy omena (Silver Cyprinid) and feed your family. The money you have is what will determine what you feed them*” (Male FGD Respondent, Kisumu).

*“Age is a factor. For example, the elderly… they cannot eat githeri… some might not be able to chew meat. So here you have to think carefully what can suit them. But the youth are not limited to these things”.* (Female FGD Respondent, Homabay).

It was interesting to note that majority of the participants were of the opinion that the choice of food was dependent on the person’s gender. The women were of the opinion that most men preferred traditional staple food like cassava and *ugali* (maize meal) while the women settled for what they considered to be lighter meals- rice and chips.

“…*you realize that there are those foods that ladies like as opposed to men. Like sometimes I’ll get an opportunity to go to the hotel with even my female colleagues. While they would prefer even eating foods like maybe chips and sodas, most of us men would prefer eating other foods like maybe “ugali” … And I’ve realized that ladies, the majority of ladies are the ones that like the snacks, those fast foods. That is my own opinion”* (Agricultural Officer, IDI Respondent, Homabay).

### Household food staples

A majority of the participants especially in Kisumu mentioned local vegetables (e.g. sukumawiki). Other popular food items included: *Omena* (Silver cyprinid) boiled maize and beans, and *ugali* (maize meal) as staples in households. This monotony would be broken by beans, eggs, rice, or beef. The reasons mentioned by participants as to why these particular foods were preferred include affordability, perceived nutritive value, religion, satiety, medical reasons, and personal preferences. These responses were also consistent with their responses about what people in the community, in general, would normally eat.

*“… As for my family, we cannot take meat. We prefer the beans and the others. We attend the SDA church and are encouraged to eat that.” (*Male FGD Respondent, Homabay).

*“I really like traditional vegetables because I get satisfied whenever I take them, there are some nutritional benefits that our bodies gain whenever we take that food, that is why I like taking traditional vegetables”.* (Female FGD Respondent, Kisumu).

### Frequency of food purchase

Responses on the frequency of food purchases varied from participant to participant. For some, a weekly budget for the dry goods (cereals, flour) and daily purchase of perishable goods such as milk and vegetables was more feasible. Only a few suggested that they purchase foodstuff once a month. The majority of participants however reported making these purchases daily. Reasons provided for the daily purchase of food included: the need to ensure the family eats fresh food, a daily wage that only allows one to spend what is earned daily, and a lack of cold storage facilities (refrigerators).

*“Because I can’t say that I get money to buy the food for one week. At times I can get like one hundred shillings, I buy breakfast. Maybe I can buy sugar and mandazi(doughnut) for the children to eat. Lunch hour, I can get vegetables and maybe buy supper too. For me to get money to buy food for one month, is hard”.* (Female FGD Respondent, Homa bay).

### Foods for special occasions

For most families, special occasions include Christmas, when a child has done well in school when the family has guests or there is a family celebration. Meals provided on such special occasions include chapati (a round flat unleavened bread resembling naan usually made of whole wheat flour and cooked on a griddle pan) chicken, sweets, an assorted variety of store-bought baked goods, and food from the American fast food restaurant Kentucky Fried Chicken (KFC). The frequency of consumption of these foods also varied among participants with some quoting a weekly routine, others once every month, and others once or twice a year.

*“When the Lord bless me then I can cook chapatti with chicken, and the children are always happy because it is rare to get chicken in these areas.” (Female FGD Respondent, Kisumu)*

*“That day I can bring them a cake, I go to the supermarket and buy cake, yogurt, milk, and such nice things for them to be happy that day.” (FGD Male Respondent, Homa bay)*

### Change in foodscape over time

Both FGD and IDI participants stated that the choice of food and even its source had changed over time. One of the common intrapersonal level influences mentioned was convenience. Due to the nature of work, people are left with little time to prepare food and opt for store-bought options.

***“****It has changed a lot, we have left the natural food, people have started preferring the readymade food … because people have no time to try and settle in a place and say I want to grow(plant crops… and people don’t want even to go and do the sourcing for that food from where it is, people want to get ready meals and that’s why they use hotels, they go to eating places than preparing foods alone in their homes”* (Partner coordinator, IDI Respondent, Homabay).

### Community influences

#### Sources of food

Location played a major role in the participant’s responses as to where they got their food. A clear dichotomy was discernable regarding sources of food between the two sites. In Homa Bay, a majority of the participants indicated they consumed food from their farms, including a variety of cereals, legumes, root tubers, vegetables, fruits, poultry, and dairy products.

*“Things like vegetable, pumpkin leaves we get from the farm… even things like eggs, chicken… we can get something small from the farms.”* (Female FGD Respondent, Homa bay).

This was in contrast to Kisumu where most participants reported that they get their food from an open market, small local retail stores (*kiosks*), and supermarkets.

*“It can happen that Kibuye (open market) is far and you are in a hurry. You go to Obunga to a kiosk here instead of going to Kibuye, I take maybe at the kiosk some sugar. On the side of vegetables, I go to a stall, I take Sukuma (local green vegetable) or omena (small endemic fish) or tomatoes.” (*Female FGD Respondent, Kisumu).

### Household food staples

Other community level influencers mentioned by the participants include Available foods in the market, regional staple foods, seasonality of produce, convenience,

*“…. it comes a time when there are no Irish potatoes may be because the areas that plant it does not have it…and the Omena also have a season from April to July, towards the end when the water is very cold, and they are not available. You will find that there are some species like Tilapia that are not found or are very few, so you will find that there will be a change in the type of food depending on the circumstance.”* (Fisherman, IDI Respondent, Homa Bay).

The existence of taboos about food was mentioned as a cultural/ community influence. There are some parts of the chicken that women were prohibited from eating. In addition, mothers-in-law are not supposed to eat chicken in their son-in-law’s house as a sign of respect.

*“.. There are some foods that are… taboos that are associated with food. Like some people in the community, they may say women are not supposed to eat eggs and even to eat chicken so those are taboos but they are not written”* (Public Health Officer, IDI Respondent, Homabay).

Some participants were of the opinion that food choice is also influenced by individuals wanting to be associated with a particular social class and wanting to fit in, therefore choosing to eat foods considered ‘classy’ even though they sometimes struggle to afford them.

### Food purchasing and preparation

Gender roles in the community played a major role in food purchase and preparation. Although some participants on both sites mentioned that both the man and woman participate in the purchase of food, the majority agreed that the women were solely responsible. Their reasons for this also varied.

*“…the man wouldn’t know the whole budget. He can buy vegetables and fail to buy tomatoes. Again for me, on the same amount of money, I may notice the baby may need fruits even if it is 5/-* ~ *( USD 0.05)- and maybe he won’t be able to remember something like that”.* (Female FGD Respondent, Kisumu).

*“As for me, this issue why we like to give them (wives) is because of cooking, they are the ones who know how they schedule the menu, so you cannot force them to cook the food she did not want, because if she decides on her own, then she will cook it nicely… we do not like buying…She is the one who knows how to coordinate what food to be eaten in her house, you know, that today I want to cook githeri ( a mix of maize and beans), tomorrow I want chapati, so she is the one who knows how she runs the house, so you cannot just do things your way, so matter food, you leave to her”. (*Male FGD Respondent*,* Kisumu).

The majority of the participants were of the opinion that it was the women who prepared meals in the home. Some of the reasons cited include the working hours of the man of the house and traditional expectations.

“*Most of us agree here it is the wife who cooks. I know how to cook, but it is just known she is the one who makes meals for us… you also have to remember we are away from the home most of the day at work, so it is easier when she is the one preparing meals”.* (Male FGD Respondent, Kisumu).

Some participants also observed that both men and women were involved in the cooking while others cited older children lending a hand in preparing meals.

*“In cooking, the children cook, I also cook and my wife also cooks. Because there are children who have grown up and have learned the art of cooking, and perhaps we may go on a journey like a funeral at my in-laws, will the children sleep hungry? I have taught them how to cook.”* (Male FGD Respondent, Homa bay).

### Change in foodscape over time

Participants mentioned the change in the physical environment as a major source of concern. Climate change has affected the seasons making it difficult for farmers to plan planting seasons. This has also affected the production of fish in the lake.

*“.. It has already changed and will continue to change. Right now, the rains have become unreliable for a while… The harvests have not been good for a while. Even the fishermen say there is less fish in the lake these days,…that’s why you hear of fish cages these days…we are also going for food which is already canned in the markets, in the supermarkets so our sources of food will definitely change we’ll go for industrial, industrially manufactured food instead of farm-produced food so it will change.” (* Officer in the Ministry of Water, IDI Respondent, Homabay).

### National/ macro-level influences

#### Choice of food and food staples

The majority of the participants from both IDIs and FGDs mentioned distance to food markets as a major determinant of what people ate in households. This especially stood out from participants from Homa Bay.

***“**** Yes, there are a number of people who travel to get food because most towns are not food sufficient, if they have cereals, they don’t have the greens if they have the greens they don’t have the cereals so they are forced to travel to get what they don’t have … families along the lake will have fish but they will not have the cereals and the ones in the upper regions will have cereals but will not have the fish so they are forced to travel to sell or travel to obtain food which they do not have.” (*NEMA officer, Homabay).

Other factors mentioned as influencing the choice of food at the macro level include Food prices, political instability, health education, and road infrastructure.

*“Then interaction with other people will also influence what people take like, especially for mothers who visit health facilities they would be taught on how to feed their children that to some extent also influences what they give to their children*.” (Nutrition Officer, Homabay).

*“…you can just imagine if somebody wanted to eat fish, and you know the majority of fish comes from the Suba region and because of the impassable roads, it was not easy for this particular fish to reach here. But now you go to the markets where you find fish fresh from ahh fresh fish from Mbita is able to reach here earlier because of the good roads that we have here.”* (Agricultural Officer, IDI Respondent, Homabay).

It was noted that the export of locally available food e.g. fish led to a hike in prices of the product in the area of origin.

**“** …*Homabay county is surrounded with the lake and its the main source of income for them… if the lake is the main source and a good number of people have come to take advantage over them and you find the bigger fish like Tilapia, Nile perch, some kind of bigger fish… they are being transported out.. what remains here you cannot even afford for your family”*

### Change in foodscape over time

Participants were concerned about overpopulation and the lack of urban planning that has, in their opinion affected food security. This has in turn shifted the foodscape from traditional wet markets to refined foods in the supermarket.

***“**** We are in a society which is ever overpopulated which is moving very faster at the higher growth rate but there’s no planning, physical planning for scarce resources… people will be competing as we compete with the huge population coming up, food depletion is there, food preference will change like that because of scarcity yeah and because of the population growth so you, I am telling you there are people who are not taking even omena, fish but because of the high demand of the population demand, and the scarcity of food, they have decided to go and even take even “mbuta” people were not taking it but nowadays they are taking so I am saying it this way, because the production will be low from the source, and the demand is high, people rush to the artificial food which is readily available like go for meals which sometimes become scarce, sometimes it becomes scarce and the prices go up you find that somebody will just go to the supermarket and pick whatever is there and forget about actually they will even say that even me, I have never been doing farming even for the last four years, maybe five just because I prefer buying which is ready.” (*Partners Coordinator, IDI Respondent, Homabay).

A participant cited improved road networks as an influencing factor in the change of diet in many households. The food not produced in various towns is easily distributed to other areas in demand.

**“** *Now, I will agree that kind of there’s a little change still because of the access of now more vegetables coming in and we have a road now from Kisii which has shortened kind of business so you find people coming to Homabay which is a central place of population, so this coming of vegetables and then we have the issue of greenhouse and then the planting of vegetables along the lake and then there’s a challenge that has been given in Rachuonyo that has triggered people planting vegetables at least nowadays you can find Omena and kales on the table, earlier on it was Omena, Omena, Omena, kales was just like a privilege to be put on the table, yes.” (Education* Officer, IDI Respondent, Homabay).

## Discussion

This qualitative study provides a useful perspective on the relationship between food retail and dietary preferences across various levels of influence in Kisumu and Homabay Counties in Western Kenya. Consistent with other findings [23, 24]. The results of this study demonstrate that the influences on dietary preference and local foodscapes are multifaceted. While public health interventions aimed at changing dietary patterns often focus on healthy food choices and increasing nutritional knowledge, the complexity of how people select their food adds weight to the assertion that shaping the food environment has the potential to support healthful eating decisions [25].

Evidence-informed approaches are increasingly prominent on national agendas for health policy and health research especially in LMICs in relation to NCDs [26]. This shift is partly in response to the high incidence of diabetes, high blood pressure, and obesity in these settings, a phenomenon linked to poor diet and nutrition. This study contributes to the evidence within various disciplines that suggests that food choice is influenced by environmental, individual, and behavioral factors.

### Interpersonal influences

Although this study stratified the focus group discussions by socioeconomic status, which is a major inter-personal influence on food choice [27], this was not demonstrated in this study. This could have reflected a true relationship, as shown in other studies [28], but may also be at least partially related to the potential misclassification of household SES [28]. Using judgments of Community health workers on socioeconomic status may have potentially biased the sample towards middle socioeconomic status households thus giving a biased sample frame [19].

Other interpersonal influences such as perceived satiety experienced with some foods in comparison to others, cost of certain foods, and transportation costs – all influenced participants’ choice and source of food. This is consistent with findings from other studies [29]. The majority of participants in our study described some foods as ‘light weight’ thus contributing to overreliance on starch dense foods to sustain individuals much longer. This was partly associated with the nature of work. Although it is widely believed that the urbanization phenomenon is largely associated with a shift in cultural dynamics [30], traditional foodscapes, and an increase in unhealthy food [31], it is noteworthy that participants in both sites cited the preference for local indigenous vegetables and locally available fish species as a staple with only an occasional indulgence in highly processed foods during special occasions. This could be attributed to the culture of the people, the vibrant fishing industry, and the perceived lack of satiety from fast foods. The decision to eat fast foods was also noted in other studies [32] and could also be looked into more as a community influence where special occasions and socializing are associated with increased consumption of processed food and drinks.

### Community influences

In our study, social pressure was seen as a barrier to healthy eating with participants mentioning that wanting to fit in would force others into unhealthy eating habits. This was in part in line with a study conducted in Germany [33] among campus students that found different views with regard to social aspects. While some participants felt that a positive peer group including family and friends steered them toward healthy eating habits, others on the other hand saw this as a barrier. Although this can be seen as a community-level influence, results from this study provide a glimpse of the role of the social networks in influencing food choice. This information can be used to better design health interventions that promote self-efficacy or encourage more family-based healthy eating promotional activities.

The local-based food pattern of *ugali* and some types of fish was defined as one of the key influencers of diet in this study suggesting that even with the county at a nutritional transition, regional staple foods are still popular. This was similar to findings by others [34]. Public health campaigns could use this information to promote locally produced food options to increase levels of uptake of healthier choices with targeted marketing.

### Macro-level influencers

The high price of food was iterated by both FGD and IDI participants as a major influence on dietary preference. These findings were consistent with other studies [35–37]. This study reveals that the choice of food goes beyond personal preferences of taste and satiety but is also strongly influenced by the economic environments that determine what food is available and at what cost. The rise in the cost of food, as well as the challenges of accessing it because of transport costs, was mentioned by participants as an example of a major influence at the macro level. There is a need for government to evaluate the price structures. This could potentially be done by reviewing taxation policies or providing subsidies, especially for staple and healthy food options.

With participants in this study worrying about the chemical content and fertilizer in the groceries sold in wet markets, there is a demand for policies that protect the food supply through the protection of the natural environment. These could include the prevention of industrial contamination of food and water, which could have other potential macro-level impacts on opportunities for healthy eating.

### Study limitations

This study is one of few qualitative investigations into food choices and practices in this context. However, this study was not without limitations. As described, CHV judgments on socioeconomic status may have biased the sample towards lower and middle socioeconomic status households; greater diversity in the social-economic status of the participants may have provided additional insight. Though efforts were made to stratify focus groups in such a way to promote frank discussion (e.g. males separate from females), it still may be that social factors prohibited the discussion of some topics or the expression of opinions perceived to differ from the norm. To further qualify the responses by participants, it would be beneficial to include a quantitative assessment of daily food consumption in households since studies have shown significant variations in reported dietary intake as compared to actual consumption. In addition, to fully appreciate the multifaceted nature of the influencing factors in dietary preference, future studies, especially in Africa, would need to incorporate detailed views of the participants with regards to cultural influences, family dynamics, and political influences that were not fully explored in this study but have acted as a backdrop to the responses from the participants.

## Conclusion

In conclusion, this study demonstrated that dietary preferences are complex and require interpretation through many lenses. Future interventions should not only consider intrapersonal and interpersonal influences when aiming to promote healthy eating among these communities but also need to target the community and macro environments. This means that nutrition promotion strategies should focus on multiple levels of influence that broaden options for interventions. However, government interventions to address food access, affordability, and marketing remain essential elements of any significant change.

## Supplementary Information

**Additional file 1: Appendix 1.** Resident Focus Group Guide- Kisumu and Homabay.

**Additional file 2: Appendix 2. **In depth Interview discussion guide.

**Additional file 3: Supplimentary file. **Themes and Codes.

## Data Availability

The focus group discussion guides used in this study are provided as supplementary documents. The transcripts generated for the FGDs will be availed upon request. The corresponding author will be available to provide these and any additional information required.

## References

[1] Cornil Y, Chandon P (2016). Pleasure as an ally of healthy eating? Contrasting visceral and Epicurean eating pleasure and their association with portion size preferences and wellbeing. Appetite.

[2] Global Burden of Disease Collaborative Network (2012). Global Burden of Disease Study 2010 (GBD 2010) Results by Cause 1990–2010.

[3] World Health Organization: European Food and Nutrition Action Plan 2015–2020Regional Committee for Europe 64th session Copenhagen, Denmark, 15–18 September 2014. URL: 64wd14e_FoodNutAP_140426 (who.int) Accessed on 12 Jan 2022.

[4] World Health Statistics: World Health Organization (https://www.who.int/gho/publications/world_health_statistics/EN_WHS2011_Full.pdf.) Accessed on 24 Apr 2021.

[5] Global action plan for the prevention and control of noncommunicable diseases 2013–2020. URL: 9789241506236_eng.pdf (who. int) Accessed on 20 Jan 2022

[6] Cecchini M, Sassi F, Lauer JA, Lee YY, Guajardo-Barron V (2010). Tackling of unhealthy diets, physical inactivity, and obesity: health effects and cost-effectiveness. Lancet.

[7] World Health Organisation (WHO) (2011). Global status report on non-communicable diseases.

[8] Maiyaki MB, Garbati MA (2014). The burden of non-communicable diseases in Nigeria; in the context of globalization. Ann Afr Med.

[9] Cohen N (2018). Feeding or Starving Gentrification: The Role of Food Policy, policy brief.

[10] Moore R. Understanding ‘gentrification’ in Southeast and East Asia. Interdisciplinary Studies. 2013;13.

[11] Nations U. Transforming our world: the 2030 agenda for sustainable development. New York: United Nations, Department of Economic and Social Affairs; 2015.

[12] Turner C, Aggarwal A, Walls H (2018). Concepts and critical perspectives for food environment research: A global framework with implications for action in low- and middle-income countries. Glob Food Sec.

[13] Foley L, Francis O, Musuva R, Mogo ER, Turner-Moss E, Wadende P, Were V, Obonyo C (2020). Impacts of a New Supermarket on Dietary Behavior and the Local Foodscape in Kisumu, Kenya: Protocol for a Mixed Methods, Natural Experimental Study. JMIR Res Protoc..

[14] Fitzgerald N, Spaccarotella K (2009). Barriers to a Healthy Lifestyle: From Individuals to Public Policy — An Ecological Perspective. Journal of Extension..

[15] Republic of Kenya (RoK). Kenya Population and Housing Census 2019, Volume II; Distribution of Population by Administrative Units. Kenya National Bureau of Statistics; 2019.

[16] Were V, Foley L (2022). Comparison of household socioeconomic status classification methods and effects on risk estimation: lessons from a natural experimental study, Kisumu, Western Kenya. Int J Equity Health.

[17] Ministry of Health Kenya (MoH) 2019: Primary Health Care Strategic Framework 2019–2024

[18] Muga R, Kizito P, Mbayah M, et al. Overview of the health system in Kenya. Kenya service provision assessment (KSPA 2004) survey

[19] Were V, Foley L, Turner-Moss E, et al. Comparison of household socioeconomic status classification methods and effects on risk estimation: lessons from a natural experimental study, Kisumu, Western Kenya. Int J Equity Health. 2022;21(1):47. 10.1186/s12939-022-01652-1 PMC8994881 35397583

[20] O’reilly M, Parker N (2013). ‘Unsatisfactory Saturation’: a critical exploration of the notion of saturated sample sizes in qualitative research. Qual Res..

[21] Graebner ME, Martin JA, Roundy PT (2012). Qualitative data: Cooking without a recipe. Strateg Organ.

[22] Campbell JL, Quincy C, Osserman J, Pedersen OK (2013). Coding in-depth semistructured interviews: problems of unitization and intercoder reliability and agreement. Sociol Methods Res..

[23] Hardcastle SJ, Thøgersen-Ntoumani C, Chatzisarantis NL (2015). Food Choice and Nutrition: A Social Psychological Perspective. Nutrients.

[24] Trübswasser U, Verstraeten R, Salm L (2021). Factors influencing obesogenic behaviors of adolescent girls and women in low- and middle-income countries: A qualitative evidence synthesis. Obes Rev.

[25] Lake A (2018). Neighborhood food environments: food choice, foodscapes, and planning for health. Proc Nutr Soc.

[26] Gaihre S, Kyle J, Semple S (2019). Bridging barriers to advance multisector approaches to improve food security, nutrition and population health in Nepal: transdisciplinary perspectives. BMC Public Health.

[27] Allen L, Williams J, Townsend N (2017). Socioeconomic status and non-communicable disease behavioral risk factors in low-income and lower-middle-income countries: a systematic review. Lancet Glob Health.

[28] Kabudula CW, Houle B, Collinson MA (2017). Assessing changes in household socioeconomic status in rural South Africa, 2001–2013: a distributional analysis using household asset indicators. Soc Indic Res.

[29] Gastón AresLeandro, Machín Alejandra Girona María Rosa Curutchet Ana Giménez. Comparison of motives underlying food choice and barriers to healthy eating among low medium income consumers in Uruguay. Cad. Saúde Pública 2017; vol.33 no.4. 10.1590/0102-311X00213315 28538799

[30] Seto KC (2016). Navin Ramankutty Hidden linkages between urbanization and food systems. Science.

[31] Battersby J, Watson V (2018). Addressing food security in African cities. Nature Sustainability.

[32] Caperon L, Arjyal AKCP, Kuikel J, Newell J, Peters R (2019). Developing a socio-ecological model of dietary behavior for people living with diabetes or high blood glucose levels in urban Nepal: A qualitative investigation. PLoS ONE.

[33] Hilger-Kolb J, Diehl K (2019). ‘Oh God, I Have to Eat Something, But Where Can I Get Something Quickly?’—A Qualitative Interview Study on Barriers to Healthy Eating among University Students in Germany. Nutrients.

[34] Lim RBT, Wee WK, For WC, Ananthanarayanan JA, Soh YH, Goh LML, Tham DKT, Wong ML (2019). Correlates, Facilitators, and Barriers of Healthy Eating Among Primary Care Patients with Prediabetes in Singapore—A Mixed Methods Approach. Nutrients.

[35] de Mestral C, Stringhini S, Marques-Vidal P (2016). Barriers to healthy eating in Switzerland: A nationwide study. Clin Nutr.

[36] Mc MorrowLudbrook LA, Macdiarmid JI, Olajide D (2017). Perceived barriers towards healthy eating and their association with fruit and vegetable consumption. J Pub Health.

[37] Farahmand M, Amiri P, Ramezani F (2015). What are the main barriers to healthy eating among families? A qualitative exploration of perceptions and experiences of Tehranian men. Appetite.

