# Supplementary material for: Navigating the local foodscape: qualitative investigation of food retail and dietary preferences in Kisumu and Homa Bay Counties, western Kenya
Source: BMC Public Health. 2022 Jun 14;22:1186. doi: 10.1186/s12889-022-13580-4 (PMC9199252; doi:10.1186/s12889-022-13580-4)
Supplement: Supplementary file 1 — Additional file 1: Appendix 1. Resident Focus Group Guide- Kisumu and Homabay. [file 12889_2022_13580_MOESM1_ESM.docx]

**Work Package 2: Resident Focus Groups**

**Introduction**

Good morning/afternoon/evening. My name is [….] and I am collecting data on behalf of researchers from the Kenya Medical Research Institute and Kisii University in Kenya, and researchers from the University of Cambridge in the United Kingdom. Previously we attended your household to collect information for a study on food retail in Kisumu/ Homabay. We would like to understand more about where households buy food and what they eat, as well as what types of food outlets are available in the local area. We are particularly interested in this new hypermarket that is opening soon at Lake Basin Mall in Kisumu. In addition we want to understand more about the relationship between eating habits and health. You gave permission for us to contact you again regarding other parts of the study. We are now conducting focus groups in your local community that will take about two hours in total. Would you be willing to hear more about this extra part of the study?”

- If yes, proceed to use the consent form

**Topic A: Food Source**

**In the first part of our discussion, I would like us to talk about where we get the foods we usually eat.**

1. Where do you usually get your food?

**Probe for**

- - - Why would you choose these sources to get the foods you need for your family

1. Who gets the food for the family?

**Probe for**

- - - Do they get the food alone or with others?

1. How do you travel to get food (i.e walk, drive, public transport)

**Probe for**

- - - If they use public transport/boda, how much do they spend
    - If they walk/drive , how long does it take

1. Now, thinking about only the food you buy, where do you usually get this food from?

**Probe for**

- Are there different types of food you buy from different places?
- What determines your choice of those places?

1. How often do you buy food?
2. Who USUALLY pays for the food?

**I would now like us to talk about your food preferences and habits as well as those of your family**

1. Can you tell me the foods that are often consumed in your **home**?
2. Can you tell me the foods that are often consumed in your **community**?
3. What are some of the foods you treat yourself or your family to?
4. You have shared with me some of the foods that are usually / often consumed in your home. Why do you prefer to eat those particular foods and not others?
5. With whom do you usually share your meals with?
6. Where do you usually eat your meals?

**Probe for**

- On normal days
- On special occasions

1. How do you store the food in your home?
2. What cooking methods are most commonly used in your home?

(deep frying, grilled, boiled etc)

1. Who usually does the food preparation and cooking in your home?

**We will now have a discussion about health and well being**

1. What foods do you consider healthy?
2. Where can you get these foods from?
3. What kind of food preparation/ cooking makes them healthy?
4. Are you concerned about the health of your **family**?

**Probe for**

- **If yes, Why? If no, why?**

1. Are you concerned about the health of your **community**?

**Probe for**

- If yes, why? If no, why?

1. Have you heard about the supermarket at the lake basin mall in Kisumu?

**Probe for**

- If yes, what have you heard?
- What kinds of items are you looking forward to purchasing from it?

**Hypermarket and Foodscape Study: Resident Focus Group Guide**

**(Homa Bay)**

- Collect socio- demographic information

**Introduce the FGD:**

- **This is an FGD with (Women/Men )**
- **Venue ( tourist hotel etc )**
- **The moderator in this FGD is..**
- **The note tacker in this FGD is..**
- **The time now is..**
- **Get verbal consent**

**Topic A: Food Source**

**In the first part of our discussion, I would like us to talk about where you get the foods you usually eat.**

1. Where do you usually get your food?

**Probe for**

- - - Are there different types of food you buy from different places?
    - Why would you choose these sources to get the foods you need for your family

1. Who gets the food for the family?

**Probe for**

- - - Do they get the food alone or with others?
    - Why are they the ones who get the food?

1. How do you travel to get food (i.e walk, drive, public transport)

**Probe for**

- - - If they use public transport/boda, how much do they spend
    - If they walk/drive , how long does it take

1. How often do you buy food?

**Probe for**

- Which types of food
- (Daily, every other day, weekly, monthly etc)

1. Who USUALLY gives the money for the food?

**I would now like us to talk about your food preferences and habits as well as those of your family**

1. Can you tell me the foods that are often consumed in your **home**?

**Probe for**

- Why they prefer to eat those particular foods and not others

1. Can you tell me the foods that are often consumed in your **community**?
2. What are some of the foods you treat/ reward yourself or your family to?
3. You have shared with me some of the foods that are usually / often consumed in your home. Why do you prefer to eat those particular foods and not others?
4. Where do you usually eat your meals? **Responses on eating at home or eating out**

**Probe for**

- On normal days (typical weekday and weekend)
- On special occasions

1. How do you store the food in your home?

**Probe for**

- Where they store different kinds of food

1. What cooking methods are most commonly used in your home?

**(deep frying, grilled, boiled, pan fry, roasting etc)**

1. Who usually does the food preparation and cooking in your home?

**Probe for**

- Why that individual does most of the cooking

**We will now have a discussion about health and well being**

1. What foods do you consider healthy?

**Probe for**

- What food preparation method makes them healthy?

1. Where can you get these healthy foods from?
2. What worried you about the health of your **family**?
3. What worries you about the health of your **community**?
