# Supplementary material for: Navigating the local foodscape: qualitative investigation of food retail and dietary preferences in Kisumu and Homa Bay Counties, western Kenya
Source: BMC Public Health. 2022 Jun 14;22:1186. doi: 10.1186/s12889-022-13580-4 (PMC9199252; doi:10.1186/s12889-022-13580-4)
Supplement: Supplementary file 2 — Additional file 2: Appendix 2. In depth Interview discussion guide. [file 12889_2022_13580_MOESM2_ESM.docx]

**Hypermarket, Foodscape and Health Study**

**Stakeholder semi structured interview**

****Remember to fill in the sociodemographic information**

**Introduce the interview:**

- **This is an interview with (title of interviewee e.g County Director for Trade, Agriculture, Education, Urban Planning, or Religious leader)**
- **Venue ( his/her office, other preferred venue)**
- **The moderator in this interview is..**
- **The note taker in this interview is..**
- **The date and time now is..**
- **Get verbal consent**

**I would like to begin this discussion by talking about the issue of food and development in Homa Bay**

1. What foods do people commonly eat in Homabay?

**Probe for:**

- What influences food choice
- Does food choice differ by Ages
- Does food choice differ by sex
- Does food choice differ by Social economic status
- Has the choice of food changed over time
- How might the choice of food change in future

1. Where do people currently get their food from in Homa Bay?

**Probe for:**

- Do they have to travel to get food? (how many mins walk?/ how many mins drive?)
- Where do people get different food types?
- Does where people get food differ by age
- Does where people get food differ by Social Economic classes
- Does where people get their food changed over time?
- How might where people get their food change in future?

1. What factors in your opinion are driving development?

**Probe for**:

- What factors drive Economic development
- What factors drive development in the Health sector
- Who holds power in influencing/ making these decisions?

1. In what ways are development plans and decisions for Homabay considerate about the impact on environment and health of the people?

**Probe:**

- Availability of healthy foods/ inequalities?
- Impact assessments on environment and health ?

1. How have you been involved in the development of supermarkets and food outlets in Homa Bay?l

**Probe for**

- How have you been involved the Food supply chains
- If no, how would you have liked to be involved?
- If yes, how else would you have liked to be involved?
- What other groups/individuals are usually involved

**Next, I would like to us to discuss the role of supermarkets in health and development**

1. Can you tell me about the location of the largest supermarket in Homa Bay?

**Probe for**

- What are the features around the supermarket? (roads, security,maket near by)
- Distance from town center

1. Why do you think the largest supermarket in Homa Bay was situated where it is and not anywhere else?
2. Who do you think are the major customers for this large supermarket in Homabay?

**Probe for:**

- Which age group mostly goes there
- Which gender mostly goes there
- What social economic status go there the most

1. What do you see as the impact of this supermarket?

**Probe for**

- Impact of the supermarket on locals
- Impact of the supermarket on investors,
- Impact of the supermarket on the environment
- The general Positive and Negative impacts and mitigation strategies
- Impact on economy of Homa bay town
- Impact on the health of the locals

1. Do you see the largest supermarket in Homabay as an income generating strategy for you or the locals?

**Probe for**

- In what way?
- Income generated 1) local people 2) County level 3) National Level

1. What do you think might be the impact of setting up a hypermarket in Homa Bay

**Probe for**

- Negative and positive impacts
- Impact on Price, variety of foods, availability of foods
- Impact on the health of the local people
- Impact of the supermarket on the number or type of outlets
- Impact of the supermarket on Particular outlets that shoppers might switch from
- Impact of the supermarket on Informal vendors

1. Which other stakeholders do you think I should include in the interviews, and why?
2. Before we close this discussion, is there anything you would like to add with regards to the **food environment and role of the big supermarkets in Homa Bay, regarding food sources and/or the health of the people of Homa Bay?**

**We have come to the end of the discussion. Thank you very much for your time and valuable insights**
