# Supplementary material for: Navigating the local foodscape: qualitative investigation of food retail and dietary preferences in Kisumu and Homa Bay Counties, western Kenya
Source: BMC Public Health. 2022 Jun 14;22:1186. doi: 10.1186/s12889-022-13580-4 (PMC9199252; doi:10.1186/s12889-022-13580-4)
Supplement: Supplementary file 3 — Additional file 3: Supplimentary file. Themes and Codes. [file 12889_2022_13580_MOESM3_ESM.docx]

**A: Themes and codes**

| **Selected Theme and sub-theme** | **Codes under a selected theme** |
| --- | --- |
| **INDIVIDUAL FACTORS**     1. Sources of food 2. Food staples 3. Frequency of food purchase 4. Change in foodscape | Freshness of produce |
|  | Transport costs |
|  | Distance |
|  | Medical reasons |
|  | Satiety |
|  |  |
|  | Nutritive value |
|  | Satiety |
|  | Availability |
|  | Religion |
|  | Age |
|  | Gender |
|  |  |
|  | Household social economic status |
|  | Cold food storage facility |
|  |  |
|  | Convenience |
|  |  |
| **COMMUNITY FACTORS**   1. Choice of food 2. Food staples 3. Change in foodscape |  |
|  | Regional staples/ Location |
|  | Seasonal foods |
|  |  |
|  | Regional staples |
|  | Seasonal foods |
|  | Culture |
|  | Population |
|  |  |
|  | Climate change |
|  |  |
| **MACRO FACTORS**   1. Choice of food and food staples 2. Change in foodscape |  |
|  | Pricing of food |
|  | Distance from market |
|  | Political instability |
|  | Heath education |
|  | Road infrastructure |
|  | Export |
|  |  |
|  |  |
|  | Urban Planning |
|  |  |
